# Effects of tempol on renal medullary tissue hypoxia in an ovine model of Gram‐negative septic acute kidney injury

**DOI:** 10.1113/EP092396

**Published:** 2025-09-22

**Authors:** Rachel Peiris, Anton Trask‐Marino, Alemayehu Jufar, Ashenafi H. Betrie, Adam Southon, Sally G. Hood, Rinaldo Bellomo, Abraham H. Hulst, Clive N. May, Connie P. C. Ow, Yugeesh R. Lankadeva

**Affiliations:** ^1^ Preclinical Critical Care Unit, Florey Institute of Neuroscience and Mental Health The University of Melbourne Melbourne Victoria Australia; ^2^ Translational Neurodegeneration Laboratory, Florey Institute of Neuroscience and Mental Health The University of Melbourne Melbourne Victoria Australia; ^3^ Department of Critical Care, Melbourne Medical School The University of Melbourne Melbourne Victoria Australia; ^4^ Australian and New Zealand Intensive Care Research Centre (ANZIC‐RC), School of Public Health and Preventive Medicine Monash University Melbourne Victoria Australia; ^5^ Department of Intensive Care Austin Hospital Melbourne Victoria Australia; ^6^ Department of Intensive Care Royal Melbourne Hospital Melbourne Victoria Australia; ^7^ Department of Anesthesiology, Amsterdam UMC University of Amsterdam Amsterdam The Netherlands; ^8^ Department of Anesthesiology Austin Hospital Heidelberg Victoria Australia

**Keywords:** acute kidney injury, hypoxia, oxidative stress, renal microcirculation, sepsis, tempol

## Abstract

Renal arterial infusion of tempol (RAT) at the onset of Gram‐negative sepsis can prevent sepsis‐induced medullary tissue hypoxia and acute kidney injury (AKI). However, it is not known whether treatment with tempol at a clinically relevant time point of sepsis is similarly effective. Thus, we examined whether tempol can reverse renal medullary tissue hypoxia after ovine Gram‐negative septic AKI. Following right unilateral nephrectomy, the left kidney was instrumented with a renal arterial catheter and oxygen‐sensing fibre‐optic probes into the renal medulla. After 23 h of *Escherichia coli* infusion, conscious sheep were fluid resuscitated with Hartmann's solution (30 mL/kg over 0.5 h) and randomized to intravenous tempol (IVT; *n* = 7) at 30 mg/kg/h, RAT (3 mg/kg/h; *n* = 6) or vehicle (*n* = 5) from 24 to 31 h of sepsis. At 31 h, *E. coli* infusion ceased, and sheep received ceftriaxone (1 g) and were allowed a 48 h recovery period. At 23 h of *E. coli* infusion, septic sheep developed a 2.2 ± 0.8‐fold increase in plasma creatinine and a 57% ± 6% decrease in urine output, and the renal medulla was ischaemic and hypoxic. Neither RAT nor IVT attenuated the sepsis‐induced renal medullary tissue hypoxia during the 7 h intervention period. Renal medullary tissue partial pressure of O_2_ returned to the pre‐morbid levels in all groups by 16 h after treatment cessation, and sepsis was resolved with antibiotics. In conclusion, in sheep with established septic AKI, treatment with RAT or IVT did not improve renal medullary oxygenation or kidney function, in contrast to the effectiveness we have shown in early sepsis. These findings emphasize the dramatically different response to a treatment in early compared with late stages of sepsis.

## Abstract (highlights)

- **What is the central question of this study?**
  Tempol, reported to have antioxidant and anti‐inflammatory effects, has been shown to prevent development of renal medullary hypoxia and subsequent development of acute kidney injury at the onset of sepsis. We explored, in an ovine model, the potential of tempol for reversing sepsis‐induced acute kidney injury when used in a clinically relevant therapeutic window.
- **What is the main finding and its importance?**
  Tempol treatment during a clinically relevant therapeutic window was not effective at ameliorating sepsis‐induced hypoxia. Therefore, investigating alternative interventions that are effective in a relevant therapeutic window to reverse renal medullary hypoxia and acute kidney injury might be a more viable therapeutic strategy for human sepsis.

## 1 INTRODUCTION

Sepsis remains the leading cause of mortality in intensive care units worldwide (Rudd et al., 2020). Sepsis is the leading cause of acute kidney injury (AKI), which affects 50% of these patients and is accompanied by a high mortality rate and prolonged hospitalization (Liu et al., 2020). Current treatments for sepsis, including infection source control, fluid resuscitation and vasopressor therapy, with renal replacement therapy in cases of severe AKI, are largely only supportive at best. Renal medullary tissue hypoxia is emerging as a critical pathophysiological mechanism of septic AKI and its subsequent transition to chronic kidney disease (Basile et al., 2016; Zarbock et al., 2023). Therefore, therapeutic strategies that decrease renal medullary tissue hypoxia in septic AKI might have both short‐ and long‐term benefits.

Tempol (4‐hydroxy‐2,2,6,6‐tetramethylpiperadine‐1‐oxyl) is a redox‐cycling nitroxide compound. It undergoes conformational changes between the nitroxide radical and the one‐ or two‐electron oxidized oxoammonium cation, which accounts for its antioxidant and anti‐inflammatory properties and ability to increase the bioavailability of nitric oxide (Fleenor et al., 2012; Welch & Wilcox, 2001; Wilcox, 2010). Tempol has been reported to confer protection from oxidative and nitrosative stress in multiple forms of kidney diseases (Chatterjee et al., 2000; Liu et al., 2021; Luan et al., 2012). Contrary to these reports, using a clinically relevant sheep model of hyperdynamic sepsis, we previously showed that sepsis was characterized by the absence of overexpression in markers of systemic and renal oxidative stress despite the renal medulla being hypoxic (Betrie et al., 2023). In that study, we showed that renal arterial infusion of tempol (RAT) from the onset of sepsis prevented renal medullary hypoxia and the subsequent development of AKI in sheep through nitric oxide‐mediated vasodilatation, an effect not seen when tempol was administered intravenously (IVT), at a 10‐fold higher dose (Betrie et al., 2023). Together, this suggests that: (1) septic AKI can develop in the absence of renal oxidative stress; and that (2) RAT is efficacious at preventing the onset of medullary hypoxia induced by sepsis. However, the ability of tempol to reverse renal medullary tissue hypoxia in established AKI, at a time when many septic patients are treated, is unknown.

In the present study, we tested the primary hypothesis of whether RAT attenuates renal medullary tissue hypoxia when administered over a clinically appropriate therapeutic window from 24 to 31 h of established Gram‐negative septic AKI. Moreover, we aimed to investigate the effects of tempol on interleukin‐6 (IL‐6) and interleukin‐10 (IL‐10). Finally, we aimed to monitor the recovery of the renal macro‐ and microcirculation after resolution of sepsis with antibiotic therapy, with and without RAT or IVT.

## 2 MATERIALS AND METHODS

### 2.1 Ethical approval

All experimental procedures were conducted at The Florey Institute of Neuroscience and Mental Health (Melbourne, Victoria, Australia). Ethics (FINMH AEC‐19‐049) for animal experiments were approved by the Animal Ethics Committee of the Florey Institute of Neuroscience and Mental Health under the guidelines of the National Health and Medical Research Council and conformed to the ARRIVE and ARRIVE 2.0 guidelines (Percie du Sert et al., 2020). Merino ewes (1.5–2.0 years old, 40.1 ± 4.4 kg, *n* = 18) were acclimated to the laboratory environment (12 h light–12 h dark cycles) in pens for ≥1 week prior to being moved to individual metabolic cages for experimentation. Sheep were allowed free access to water and fed 800 g of oaten chaff daily.

### 2.2 Surgical preparation

All sheep underwent two aseptic surgical procedures. Antibiotic (900 mg of procaine penicillin, Ilium Propercillin, Troy Laboratories, NSW, Australia) and analgesia (1 mg/kg flunixin meglumine, Ilium Flunixil, Troy Laboratories) were administered intramuscularly at the following time points: (1) prior to the first incision of every surgical procedure; and (2) at ∼24 and ∼48 h after recovery from surgery. An additional dose of analgesia was given at ∼6 h after commencement of the surgery. Induction of anaesthesia was achieved with intravenous sodium thiopentone (15 mg/kg, Jurox Pty, Ltd, NSW, Australia) and maintained with isoflurane (2.0%–2.5% v/v isoflurane in oxygen/air).

In the first surgical procedure, the left carotid artery was exteriorized into a skin fold to facilitate arterial cannulation for measurement of the mean arterial blood pressure (MAP) and heart rate (HR) and for sampling of arterial blood in non‐anaesthetized sheep (Lankadeva et al., 2018). During the same surgical procedure, the sheep underwent a unilateral nephrectomy, whereby the right renal artery, renal vein and ureter were ligated and the right kidney was removed as previously described (Ishikawa et al., 2012). This allowed for the establishment of a one‐to‐one relationship between renal blood flow (RBF) and renal function in the remaining kidney in response to the administration of tempol via the renal artery.

After 4–6 weeks of recovery from the first surgery, the exteriorized carotid artery and jugular vein were cannulated to: (1) enable the measurement of MAP and HR and the sampling of arterial blood; (2) allow intravenous infusion of *Escherichia coli* for the induction of Gram‐negative sepsis; and (3) deliver standard care fluid resuscitation followed by infusion of noradrenaline intravenously.

One day later, the ewes underwent a second surgical procedure. In brief, following laparotomy, a Silastic catheter (internal diameter, 0.64 mm; outer diameter, 1.9 mm; Dow Corning Corporation, MI, USA) was inserted into the left renal artery to enable intrarenal infusion of tempol, and a Tygon catheter (internal diameter, 1.0 mm; outer diameter, 1.8 mm; John Morris Scientific Pty, Ltd, NSW, Australia) was inserted into the left renal vein to enable renal venous blood sampling. A transit‐time flow probe (4 mm; Transonic Systems Inc., NY, USA) was then placed around the left renal artery to measure RBF. Following this, the left kidney was exposed to allow two custom‐built fibre‐optic probes (450 µm outer diameter; CP‐0004‐0001; Oxford Optronix, Oxford, UK) to be implanted into the renal cortex and medulla (one implanted in each region). The tips of the fibre‐optic probes were advanced into the renal cortex and medulla, thereby facilitating the simultaneous assessment of renal cortical and medullary tissue perfusion, tissue oxygen tension and temperature (Calzavacca et al., 2015). The locations of the tips of the renal cortical and medullary fibre‐optic probes were confirmed at post‐mortem examination. If the tip of a probe was found to be implanted in the wrong region at post‐mortem examination or if the probe was unresponsive to the condition of the sheep prior to commencement of treatment, the data derived from these probes were not used in analysis. Lastly, a Foley catheter (14 Fr; Livingstone International Pty Ltd, NSW, Australia) was advanced into the bladder to enable later sampling of urine.

### 2.3 Experimental protocol for induction of sepsis

**FIGURE 1:**
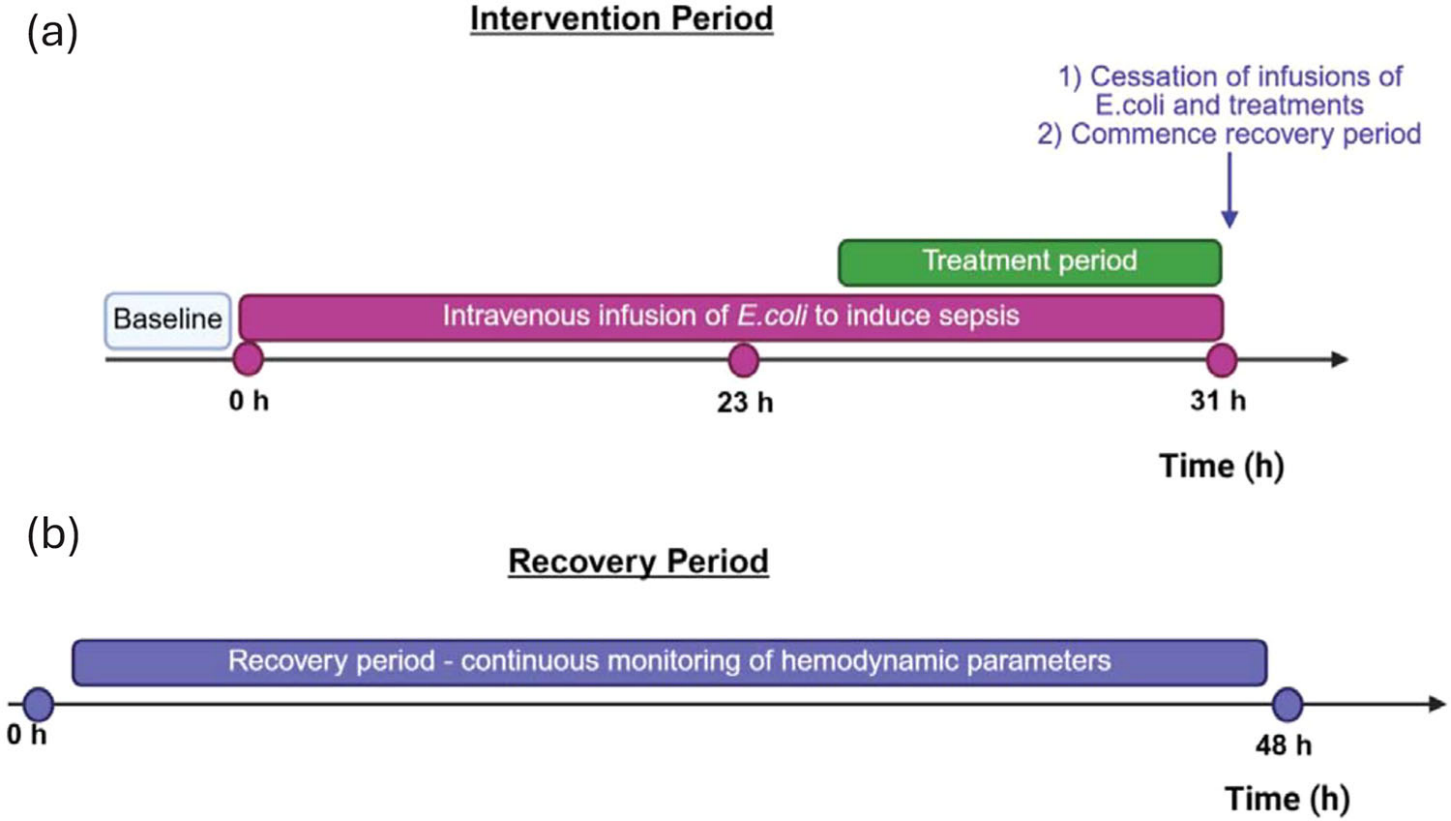
Schematic diagram of the experimental protocol. (a) After a 24 h baseline recording period, female sheep were made septic by a continuous intravenous infusion of live *Escherichia coli* for a period of 31 h. The sheep were randomized to receive one of three treatments from the 23rd to 31st hour after commencement of sepsis. (b) After this, all infusions ceased, and sheep entered the ‘recovery period’, when only continuous monitoring of haemodynamic parameters continued.

Sheep were allowed a recovery period from surgery of 3–5 days before commencement of the experiment (Lankadeva et al., 2018). After a 24 h baseline recording period, Gram‐negative sepsis was induced in conscious sheep by an intravenous infusion of a loading dose of 2.8 × 10^9^ colony‐forming units (CFU) of live *E. coli* over a 30 min period, followed by a continuous intravenous infusion of 1.26 × 10^9^ CFU/h for the next 30.5 h. All animals received standard care fluid bolus therapy of balanced crystalloid (30 mL/kg of Hartmann's solution; Baxter, NSW, Australia) from 23.5 to 24 h after *E. coli* infusion commenced. A schematic diagram of the experimental protocol is shown in Figure 1.

### 2.4 Experimental protocol for infusion of tempol

Prior to the commencement of the experiment, sheep were randomized to receive one of three treatments: (1) IVT (30 mg/kg/h; Sigma‐Aldrich, Victoria, Australia; *n* = 7, body weight = 38.4 ± 5.1 kg); (2) RAT (3 mg/kg/h, *n* = 6, body weight = 41.2 ± 4.7 kg); or (3) intravenous infusion of vehicle (6 mL/h; Hartmann's solution; Baxter Australia; *n* = 5, body weight = 41.2 ± 4.7 kg) over a 7 h period (i.e. from 24 to 31 h after induction of sepsis). The dose of tempol given for each treatment group was based on a previous proof‐of‐concept study demonstrating the efficacy of RAT at preventing sepsis‐induced renal medullary hypoxia, the primary hypothesis in this study (Betrie et al., 2023). Noradrenaline was infused intravenously from 25 to 31 h after induction of sepsis, at a dose titrated to maintain a target MAP of ∼80 mmHg.

### 2.5 Experimental protocol for recovery from sepsis

At the end of the 7 h period of infusion of tempol or its vehicle (24–31 h), all drug treatments and *E. coli* infusions ceased, and the sheep received an intravenous infusion of ceftriaxone (1 g; AFT Pharmaceuticals, NSW, Australia). Moreover, a 1 g dose of the antibiotic was also administered 24 h later. All animals received a maintenance intravenous infusion of 1 mL/kg/h Hartmann's solution throughout the recovery period. At the end of the 48 h recovery period, animals were humanely killed with a lethal dose of intravenous pentobarbitone (100 mg/kg; Lethabarb; Randlab, NSW, Australia).

### 2.6 Periodic measurements in septic sheep

Arterial and renal venous blood samples were collected at the following time points of each period: (1) pre‐morbid period, as a baseline measurement prior to induction of sepsis; (2) treatment period, at 23, 25, 27, 29 and 31 h after induction of sepsis; and (3) recovery period, at 16, 24, 40 and 48 h after initiation of the recovery period. Urine flow was measured hourly from the commencement of the treatment period and until the end of the recovery period via a modified fraction collector. Urine samples were collected at time points matched to arterial and renal venous blood samples. Arterial and renal venous blood sampling facilitated the assessment of blood gas and lactate concentration (ABL system 625; Radiometer Medical, Copenhagen, Denmark). Additionally, an aliquot of arterial blood sampled at each time point was centrifuged at 492 *g* for 10 min at 4°C in order that plasma could be collected for later analysis of renal function and levels of inflammatory markers. Concentrations of IL‐6 and IL‐10 in plasma samples were determined using commercially available kits (Kingfisher Biotech Inc., MN, USA) according to the manufacturer's instructions. Lastly, plasma and urinary concentrations of creatinine and the urinary concentration of sodium were measured at the Pathology Service at Austin Health, Heidelberg, Victoria, Australia.

### 2.7 Calculations and measurements

Analog signals of MAP, HR (triggered by the arterial pressure), RBF, renal cortical and medullary tissue perfusion, oxygen tension and temperature were acquired continuously, at 100 Hz, for the duration of the experiment using a CED interface (Micro 1401; Cambridge Electronic Design, Cambridge, UK) and recorded on Spike2 software (Cambridge Electronic Design) (Lankadeva et al., 2018). Renal oxygen delivery (RDO_2_) was calculated as the product of the arterial blood concentration of oxygen and RBF, whereas renal oxygen consumption (RVO_2_) was calculated as the product of the arteriovenous oxygen concentration difference and RBF. Renal oxygen extraction was calculated as RVO_2_ expressed as a percentage of RDO_2_. Renal vascular conductance (RVC) was calculated as RBF/MAP.

### 2.8 Statistical analysis

Normality of the data was assessed using the Shapiro–Wilk test. Data that did not violate normality are presented as the mean ± SEM, whereas data that violated normality are presented as the median [interquartile range (25th percentile, 75th percentile)]. Student's paired *t*‐test was conducted to determine whether the variable differed between the values at baseline and at 23 h of sepsis, thus assessing the severity of sepsis in response to live *E. coli* infusion. For variables measured at multiple time points, data were analysed using a two‐way repeated‐measures ANOVA, with factors treatment (*p*_treatment_), time (*p*_time_), and their interaction (*p*_treatment×time_). The *p*‐values derived from these within‐subject factors in repeated‐measures ANOVA were conservatively adjusted using the Greenhouse–Geisser method (Ludbrook, 1994). If *p*_time_ and/or *p*_treatment×time_ was ≤0.05, Dunnett's *post hoc* test was used for within‐group multiple comparisons of: (1) 23 h time point compared with 25, 27, 29 and 31 h during the intervention period; and (2) pre‐morbid baseline compared with 16, 24, 40 and 48 h during the recovery period. If *p*_treatment_ and/or *p*_treatment×time_ was ≤0.05, a Tukey's *post hoc* test was used for between‐group multiple comparisons of vehicle, RAT and IVT treatment groups at each time point. Statistical analyses and figures were generated using GraphPad PRISM software (v.10.0; GraphPad Inc., USA). Two‐sided *p* ≤ 0.05 was considered statistically significant.

## 3 RESULTS

### 3.1 Gram‐negative sepsis induced acute kidney injury

As shown in Table 1, intravenous infusion of live *E. coli* for 23 h in conscious sheep induced a hyperdynamic circulatory state, with hypotension, tachycardia, fever, increased lactate levels and global renal hyperaemia (all *p* < 0.01). Septic sheep developed Stage 2 AKI according to criteria set out in Kidney Disease Improving Global Outcomes (Bellomo et al., 2017; Work Group Membership, 2012), with a 2.2 ± 0.8‐fold increase in plasma creatinine, a 57% ± 6% decrease in urine output (*p* < 0.0001) and a 69.4% ± 6.5% decrease in sodium excretion (*p* < 0.0001). Despite the development of global renal hyperaemia, renal cortical perfusion and tissue partial pressure of O_2_ ($P_{O_{2}}$) remained unchanged. In contrast, the renal medulla developed ischaemia (−39.3% ± 15.3%, *p* > 0.01) and hypoxia (−46.5% ± 6.5%, *p* < 0.0001; Table 1).

**TABLE 1:** Systemic haemodynamics, global and regional kidney perfusion, tissue oxygenation and renal function at the baseline and 23 h after continuous infusion of live *Escherichia coli* in conscious sheep.

Parameter	Baseline	23 h sepsis
Systemic haemodynamics		
Mean arterial pressure, mmHg	87.5 ± 1.3	75.8 ± 2.2****
Heart rate, beats/min	80 ± 3	142 ± 4****
Renal blood flow, mL/min	285.2 ± 18.3	370.3 ± 30.7*
Renal vascular conductance, mL/min/mmHg	3.3 ± 0.2	4.9 ± 0.4**
Body temperature, °C	38.8 (39.3, 40.4)	41.5 (41.3, 41.8)****
Arterial blood lactae, mmol/L	0.5 (0.4, 0.8)	1.2 (0.9, 1.9)***
Indices of renal function and tissue perfusion and oxygenation		
Medullary tissue, perfusion, BPU	881 (621, 1516)	346 (225, 684)**
Cortical tissue perfusion, BPU	1652 ± 189	1573 ± 262
Medullary tissue $P_{O_{2}}$, mmHg	46.3 (38.2, 57.4)	20.8 (16.5, 25.8)****
Cortical tissue $P_{O_{2}}$, mmHg	39.7 ± 2.3	41.3 ± 3.9
Urine flow, mL/h/kg body weight	1.3 ± 0.1	0.5 ± 0.1****
Plasma creatinine, µmol/L	74 (60, 88)	131 (109, 197)****
Na^+^ excretion (mmol/min)	0.11 (0.06, 0.15)	0.03 (0.01, 0.04)****

*Note*: Normality of the data was assessed using the Shapiro–Wilk test. Data that did not violate normality are expressed as the mean ± SEM, whereas data that violated normality are presented as the median [interquartile range (25th percentile, 75th percentile)]. Dichotomous comparisons between baseline values prior to infusion of live *E. coli* and at 23 h after sepsis was induced prior to commencement of treatments were made using Student's paired two‐tailed *t*‐test. *p ≤ 0.05, **p < 0.01, ***p < 0.001 and ****p < 0.0001 of paired comparisons in all 18 sheep between 23 h sepsis and the respective baseline levels.

Abbreivation: BPU, blood perfusion unit.

### 3.2 Effects of tempol treatment on renal and intrarenal perfusion and oxygenation

**FIGURE 2:**
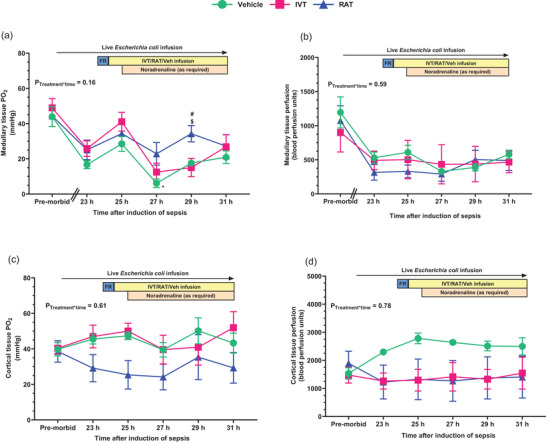
Renal tissue oxygenation and perfusion in the cortex and medulla during established sepsis, in response to 7 h treatment with either tempol or its vehicle. Renal tissue oxygenation and perfusion were assessed at the baseline, 23 h after continuous infusion of live *Escherichia coli* commenced and at four time points in the 7 h intervention period with either intravenous (IVT, *n* = 7) or renal arterial (RAT, *n* = 6) infusion of tempol or its vehicle (*n* = 5). Data are expressed as the mean ± SEM. The *p*‐values are the outcomes of treatment × time interaction from a two‐way repeated‐measures ANOVA from prior treatment at 23 h sepsis and at the four time points in the 7 h treatment period. *p ≤ 0.05 for Dunnett's *post hoc* test in vehicle‐treated sheep compared with the respective levels at the 23 h time point. ^$^ *p* ≤ 0.05 for Tukey's test comparing treatment difference with RAT treatment and ^#^ *p* ≤ 0.05 for Tukey's test comparing treatment difference with vehicle treatment.

**FIGURE 3:**
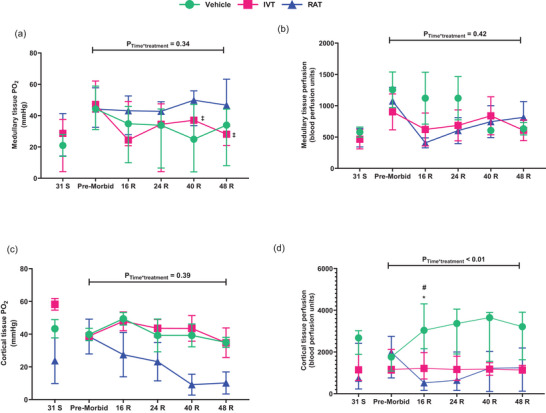
Renal tissue oxygenation and perfusion in the cortex and medulla in the recovery period following resolution of sepsis with antibiotic. At the end of the intervention period (denoted by 31 S) with either intravenous (IVT, *n* = 7) or renal arterial (RAT, *n* = 6) infusion of tempol or its vehicle (*n* = 5), sheep were given intravenous doses of 1 g ceftriaxone, a cephalosporin antibiotic, repeated at 24 h of recovery, and they were allowed to recover over 48 h. Data points at time point 31 S are included for reference to the end of the treatment period and were not included in statistical analysis. Normality of the data was assessed using the Shapiro–Wilk test. Data that did not violate normality are expressed as the mean ± SEM, whereas data that violated normality are presented as the median [interquartile range (25th percentile, 75th percentile)]. The *p*‐values are the outcomes of treatment × time interaction from a two‐way repeated‐measures ANOVA from the pre‐morbid baseline and at the four time points in the 48 h recovery period. ^*^ *p* ≤ 0.05 for Dunnett's *post hoc* test in vehicle‐treated sheep compared with the respective levels at the 23 h time point for data collected in the recovery period. ^‡^ *p* ≤ 0.05 for Dunnett's *post hoc* test in IVT‐treated sheep compared with the pre‐morbid levels for data collected in the recovery period. ^#^ *p* ≤ 0.05 for Tukey's test comparing treatment difference with vehicle treatment.

Overall, in septic sheep, RAT did not significantly attenuate the degree of renal medullary hypoxia, despite the renal medullary tissue $P_{O_{2}}$ being transiently greater compared with vehicle (*p* = 0.03) and IVT (*p* = 0.04) at 29 h of sepsis (Figure 2a). Neither treatment altered renal medullary ischaemia from 24 to 31 h of sepsis (Figure 2b). Following resolution of sepsis with antibiotics, renal medullary tissue $P_{O_{2}}$ returned to the pre‐morbid levels by 16 h of recovery in all three treatment groups (Figure 3a). However, it appears that the renal medullary tissue $P_{O_{2}}$ was significantly lower than pre‐morbid baseline levels in IVT at 40 and 48 h of recovery compared with vehicle treatment (Figure 3b). Renal cortical tissue oxygenation and perfusion did not change significantly with sepsis and were maintained at pre‐morbid levels during RAT, IVT and vehicle treatment and following antibiotic therapy (Figure 3c, d). There were no significant differences in RDO_2_, RVO_2_ and renal oxygen extraction between the treatment groups during intervention and postsepsis resolution with antibiotic therapy in all treatment groups (Tables S1 and S2). Over the 7 h intervention period, the sepsis‐induced increases in RBF and RVC persisted from 24 to 31 h of sepsis in the vehicle‐, IVT‐ and RAT‐treated sheep (Figure S1). The RBF and RVC gradually normalized to their respective pre‐morbid levels with time in all three groups by the end of the 48 h recovery (Figure S1).

### 3.3 Effects of tempol treatment on renal function

**FIGURE 4:**
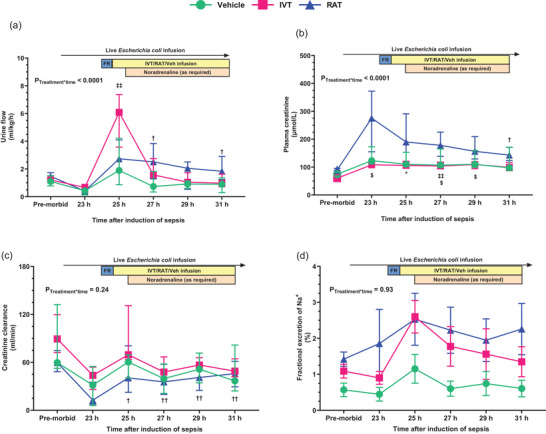
Renal function during established sepsis, in response to 7 h treatment with either tempol or its vehicle. Urine flow (a), plasma creatinine (b), creatinine clearance (c) and fractional excretion of sodium (d) were assessed at the baseline, 23 h after continuous infusion of live *Escherichia coli* commenced (23 h sepsis) and in the 7 h intervention period with either intravenous (IVT, *n* = 7) or renal arterial (RAT, *n* = 6) infusion of tempol or its vehicle (*n* = 5). Data are expressed as the mean ± SEM. The *p*‐values are the outcomes of treatment × time interaction from a two‐way repeated‐measures ANOVA from prior treatment at 23 h sepsis and at the four time points in the 7 h treatment period. ^*^ *p* ≤ 0.05 for Dunnett's *post hoc* test in vehicle‐treated sheep, ^†^ *p* ≤ 0.05 for Dunnett's *post hoc* test in RAT‐treated sheep and ^‡‡^ *p* < 0.01 for Dunnett's *post hoc* test in IVT‐treated sheep compared with the 23 h time point. ^$^ *p* ≤ 0.05 for Tukey's test comparing treatment difference with RAT treatment.

By the end of the 7 h intervention period, urine flow was significantly greater in RAT than the pretreatment levels at 23 h (*p* = 0.02; Figure 4a). At 23 h after infusion of *E. coli* before treatment began, plasma creatinine in the RAT group increased by 2.9 ± 0.5‐fold from pre‐morbid levels (Figure 4b). This increase from pre‐morbid levels was significantly greater compared with the IVT treatment group (1.8 ± 0.1‐fold, *p* = 0.03) and tended to be greater than in sheep randomized to vehicle treatment (1.9 ± 0.2‐fold, *p* = 0.06; Figure 4b). Plasma creatinine declined progressively following RAT treatment, and by the end of the treatment period it was 44.5% ± 6.3% less than at 23 h sepsis (*p* = 0.01; Figure 4b). Consequently, creatinine clearance in RAT‐treated sheep increased progressively with time and had increased by 232.3 ± 72.9 mL/min from pretreatment levels at 23 h (*p* = 0.001; Figure 4c) by the end of the treatment period. In contrast, urine output, plasma creatinine and creatinine clearance following IVT or vehicle treatment were not significantly different at 31 h compared with their respective levels at 23 h (Figure 4a–c). Finally, there were no significant changes in fractional excretion of sodium over the 7 h intervention period in all three treatment groups (Figure 4d).

**FIGURE 5:**
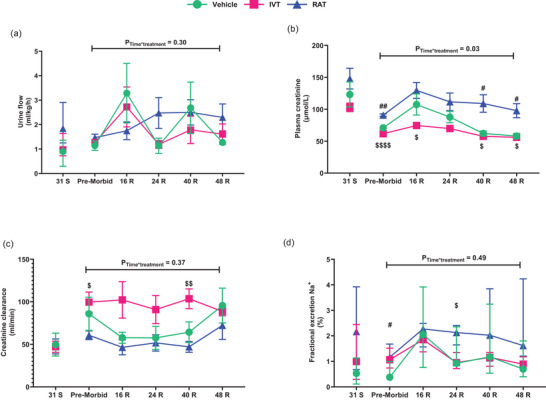
Renal function in the recovery period following resolution of sepsis with antibiotic. At the end of the intervention period (denoted by 31 S) with either intravenous (IVT, *n* = 7) or renal arterial (RAT, *n* = 6) infusion of tempol or its vehicle (*n* = 5), sheep were given intravenous doses of 1 g ceftriaxone, a cephalosporin antibiotic, repeated at 24 h of recovery, and they were allowed to recover for 48 h. Urine flow (a), plasma creatine (b), creatinine clearance (c) and fractional excretion of sodium (d) were assessed over 48 h in the recovery period. Data points at time point 31 S are included for reference to the end of the treatment period and were not included in statistical analysis. Pre‐morbid refers to the mean of 24 h of the baseline period, and the time points in intervention and recovery periods are means of 1 h periods. Normality of the data was assessed using the Shapiro–Wilk test. Data that did not violate normality are expressed as the mean ± SEM, whereas data that violated normality are presented as the median [interquartile range (25th percentile, 75th percentile)]. The *p*‐values are the outcomes of treatment × time interaction from a two‐way repeated‐measures ANOVA from the pre‐morbid baseline and at the four time points in the 48 h recovery period. Tukey's *post hoc* test was conducted when *p*_treatment_ and/or *p*_treatment×time_ ≤ 0.05. ^$^ *p* ≤ 0.05, ^$$^ *p* < 0.01 and ^$$$$^ *p* < 0.0001 for Tukey's test comparing treatment difference with RAT treatment. ^#^ *p* ≤ 0.05 and ^##^ *p* < 0.01 for Tukey's test comparing treatment difference with vehicle treatment.

Following cessation of treatment and resolution of sepsis with antibiotic, the urine flow, plasma creatinine, creatinine clearance and fractional excretion of sodium normalized to pre‐morbid levels by 16 h recovery in all three treatment groups (Figure 5). The plasma creatinine levels prior to induction of sepsis in RAT‐treated sheep were significantly greater than those in IVT‐treated sheep (Figure 5b). Therefore, by the end of the 48 h recovery period in RAT, plasma creatinine was still significantly greater than IVT‐treated sheep (*p* = 0.02) despite normalization to its pre‐morbid levels (Figure 5b).

### 3.4 Effects of tempol treatment on markers of inflammation

**FIGURE 6:**
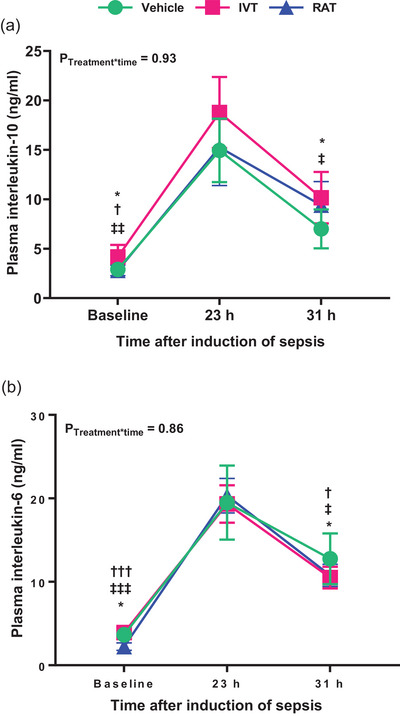
Plasma levels of interleukin‐6 and interleukin‐10 at established sepsis and at the end of 7 h treatment with either tempol or its vehicle. Plasma samples collected were analysed to determine the concentration of interleukin‐10 (a) and interleukin‐10 (b) at the baseline, 23 h after continuous infusion of live *Escherichia coli* commenced (23 h sepsis) and at the at end of the 7 h intervention period with either intravenous (IVT, *n* = 7) or renal arterial (RAT, *n* = 6) infusion of tempol or its vehicle (*n* = 5). Data are expressed as the mean ± SEM. The *p*‐values are the outcomes of treatment ± time interaction from a two‐way repeated‐measures ANOVA. ^*^ *p* ≤ 0.05 for Dunnett's *post hoc* test in vehicle‐treated sheep compared with the respective levels at the 23 h time point for data collected. ^‡^ *p* ≤ 0.05, ^‡‡^ *p* < 0.01 and ^‡‡‡^ *p* < 0.001 for Dunnett's *post hoc* test in IVT‐treated sheep compared with the 23 h time point. ^†^ *p* ≤ 0.05 and ^†††^ *p* < 0.001 for Dunnett's *post hoc* test in RAT‐treated sheep compared with the 23 h. ^$^ *p* ≤ 0.05 for Tukey's test comparing treatment difference with RAT treatment. ^#^ *p* ≤ 0.05 for Tukey's test comparing treatment difference with vehicle treatment.

In the sepsis group treated with vehicle, there were significant elevations of the pro‐inflammatory cytokine IL‐6 and anti‐inflammatory cytokine IL‐10 prior to intervention at 23 h (Figure 6). The levels of both cytokines had declined by the end of the intervention at 31 h, but the levels remained significantly greater than the pre‐morbid levels (Figure 6). There were similar changes in IL‐6 and IL‐10 in both RAT and IVT.

### 3.5 Effects of tempol treatment on systemic haemodynamic variables

During the treatment period from 25 to 31 h, with tempol or its vehicle, MAP was maintained at a target value of ∼80 mmHg with noradrenaline (Figure S2a). The dose of noradrenaline required to maintain the target MAP increased with time in all three groups (*p*_time_ = 0.006; Figure S2g). There were no significant differences in the tachycardic and febrile responses induced by *E. coli* from 23 to 31 h of sepsis between the three treatment groups (Figure S2c, e).

When noradrenaline was withdrawn after cessation of treatment and resolution of sepsis with antibiotic at the initiation of the recovery period, there was a gradual decline in MAP reaching a nadir 24 h into the recovery period in all three groups (Figure S2b). Subsequently, MAP gradually recovered and was comparable to the pre‐morbid levels by the end of the 48 h recovery period in all three groups (Figure S2b). HR remained significantly elevated at 48 h recovery relative to the pre‐morbid levels (29.6% ± 5.9%, *p* = 0.02; Figure S2d) in vehicle‐treated animals. In contrast, HR returned to pre‐morbid levels in both RAT‐ and IVT‐treated sheep at the end of the 48 h recovery period (Figure S2d). The febrile response to sepsis was ameliorated after cessation of *E. coli* infusion and with antibiotic treatment, as body temperature normalized in all three groups by 16 h after initiation of antibiotic treatment (Figure S2f).

### 3.6 Effects of tempol treatment on arterial oximetry

Sepsis‐induced hyperlactataemia was not significantly reduced by treatment. Over all four time points during the 7 h intervention period, arterial blood $P_{O_{2}}$, partial pressure of CO_2_ and oxygen saturation did not differ significantly between the IVT and RAT groups (Table S3). Arterial biochemistry variables returned to their respective pre‐morbid levels in all three treatment groups by 48 h of recovery (Table S4).

## 4 DISCUSSION

In a large mammalian model of established septic AKI, we did not observe significant improvements in renal medullary tissue hypoxia during the 7 h intervention period with IVT or RAT. Furthermore, the duration for the normalization of renal medullary oxygenation and renal function was not accelerated by tempol treatment. Therefore, tempol treatment in established sepsis was not likely to have influenced the recovery of renal medullary oxygenation after cessation of treatment and resolution of sepsis with antibiotic.

Ovine septic AKI is characterized by an early onset of renal medullary tissue hypoperfusion and hypoxia that precedes the development of AKI by 24 h. Renal medullary hypoperfusion and hypoxia occur in ovine sepsis despite an increase in global RBF and RDO_2_ and preserved renal cortical tissue oxygenation and perfusion (Calzavacca et al., 2015; Lankadeva et al., 2016, 2018). Moreover, Gram‐negative sepsis is characterized by an absence of renal tubular necrosis and apoptosis, suggesting that sepsis‐induced renal injury in ovine hyperdynamic septic AKI is functional and not structural in nature (Langenberg et al., 2014; Maiden et al., 2016). Similar findings have been reported in humans who succumbed to septic AKI, where the nature of renal injury was focal and did not explain the severe functional defects that manifested as severe oliguria/anuria and significantly elevated serum creatinine levels (Lerolle et al., 2010). Renal medullary tissue hypoxia also appears to occur in patients with sepsis and critical illness, as measured indirectly by bladder urinary oxygenation (Osawa et al., 2019; Plummer et al., 2021). These preclinical and clinical observations provide the impetus to develop strategies to attenuate renal medullary tissue hypoxia to resolve septic AKI.

We recently demonstrated that RAT treatment from the onset of sepsis prevented renal medullary tissue hypoxia and the development of AKI (Betrie et al., 2023). We found that these nephroprotective actions of tempol, when infused directly into the renal artery over 24 h of sepsis, were associated with reduced tissue inflammation in the renal cortex and improved nitric oxide bioavailability in the renal medulla (Betrie et al., 2023). However, the efficacy of tempol at later stages of infections, when interventions are likely to occur in intensive care units, has not been investigated previously to gauge its translational potential to humans with septic AKI.

In the present study, we found that neither RAT nor IVT (at 10 times the RAT dose) during established sepsis significantly attenuated renal medullary tissue hypoxia or improved renal function. In contrast, we previously reported that RAT but not IVT at the same doses used here, when given at the onset of *E. coli* infusion, ameliorated the renal medullary hypoxia (Betrie et al., 2023). In early sepsis, the disparity in therapeutic efficacy between RAT and IVT was attributed to a greater bioavailability of the superoxide‐quenching nitroxide radical form of tempol being delivered to the kidney, when tempol is infused directly into the kidney rather than when infused intravenously (Betrie et al., 2023). In early sepsis, the reno‐protective effects of RAT were associated with a greater bioavailability of the nitroxide radical than with IVT, which reduced renal tissue protein expression of tumour necrosis factor‐α and the uncoupling of endothelial nitric oxide synthase (Betrie et al., 2023). Taken together, treatment with RAT in established sepsis was not effective at improving renal function or renal tissue perfusion and oxygenation, despite the possibility of a greater bioavailability of the nitroxide form of tempol in the kidney.

We found that during the recovery period, sepsis‐induced medullary hypoxia recovered to the pre‐morbid levels by 16 h after antibiotic treatment. Likewise, previous studies in Gram‐negative septic AKI have demonstrated that the renal microcirculatory dysfunction requires 24–48 h to return to healthy physiological levels after stopping *E. coli* infusion and initiating antibiotic treatment (Calzavacca et al., 2015; Langenberg et al., 2014, 2007, 2021). In contrast, the renal macrocirculation and kidney function recovered by 16 h after initiating antibiotic treatment and stopping *E. coli* infusion. Collectively, these findings suggest that renal microcirculatory dysfunction might be sustained even when clinical indices of renal function have normalized. Future studies are warranted to explore this possibility of sustained microcirculatory dysfunction in other aetiologies of AKI, including cardiopulmonary bypass.

There are several strengths to our study. We used a clinically relevant model of sepsis, in which the phenotype closely recapitulates human sepsis characterized by a hyperdynamic circulation and the development of AKI, making it highly relevant for therapeutic translation to patients. In contrast, most rodent models of sepsis (e.g. caecal ligation and puncture, lipopolysaccharide and faecal slurry) are often characterized by a hypodynamic circulation. Our experiments were performed in conscious sheep, thus avoiding the confounding factors of anaesthesia on tissue $P_{O_{2}}$, systemic haemodynamics and renal microcirculatory perfusion (Iguchi et al., 2019, 2020). Our experiments were also conducted in accordance with the standard care that human patients receive, such as fluid bolus therapy with crystalloid and vasopressor support. Lastly, measurements of tissue $P_{O_{2}}$ were made using fluorescence‐based probes that measure dissolved tissue oxygen in the tissue volume surrounding a probe diameter of 270 µm, allowing for direct assessment of tissue oxygenation (Calzavacca et al., 2015). In contrast, other non‐invasive assessments of tissue oxygenation, such as blood oxygen level‐dependent MRI and near‐infrared spectroscopy, currently in use in human patients, quantify the proportion of blood oxygen saturation and are thus an indirect measurement of renal tissue $P_{O_{2}}$ (Ow et al., 2018).

One of the limitations in the study is the variability in the severity of AKI. The sheep randomized to receive RAT treatment had a more severe AKI characterized by a significantly greater increase in plasma creatinine from the baseline levels compared with those in the IVT and vehicle groups. Consequently, the creatinine clearance was significantly greater in IVT than in RAT. However, this could be also viewed as a strength in our study. Sheep are outbred animals and are therefore representative of population diversity and thereby representative of the heterogeneity in the sickness response to sepsis and the severity of the associated AKI, unlike inbred animal models, such as mice and rats. Another observation apparent in our study is that the renal tissue perfusion does not always represent changes in renal tissue oxygenation. This is largely because laser Doppler flowmetry in highly perfused organs, such as the kidney, measures the velocity of red blood cells and not the tissue blood flow (Eppel et al., 2003). Limitations of our study include the use of an *E. coli* strain isolated from a patient with sepsis, making our conclusions likely to be generalizable only to patients with Gram‐negative sepsis, although *E. coli* is a very common cause of sepsis, making these findings highly clinically relevant. Furthermore, the animals we used in our study were young adults and lacking the common co‐morbidities, such as old‐age, hypertension, diabetes and immunodeficiency, often associated with the development of sepsis in humans. Owing to the nature of our experiments, in which it was important to monitor the recovery from sepsis for 48 h, we could not collect tissues at the cessation of the treatment. Instead, tissue samples were collected at 48 h after recovery from sepsis. The half‐life of tempol is typically <1 h in the kidney (Wilcox & Pearlman, 2008), hence the direct effect of tempol on renal tissue was likely to have dissipated by the time of tissue sampling. Therefore, we could not assess the renal tissue for molecular markers of inflammatory and nitric oxide signalling to provide a mechanistic link to the physiological signals observed. However, given that neither RAT nor IVT had any effect during the treatment period, and there were no residual effects at 48 h recovery, it would have been unlikely that we would have observed any changes in molecular markers of inflammatory and nitric oxide signalling. A potential limitation is that noradrenaline, a first‐line clinical vasopressor, reduces renal medullary perfusion and oxygen tension for 3–4 h when used to mitigate hypotension in septic sheep (Lankadeva et al., 2016). The experimental protocol was designed to mimic clinical practice closely, thus requiring maintenance of MAP using noradrenaline as needed. As a result, we were unable to determine the effects of tempol on renal medullary tissue oxygenation without confounding effects of the transient hypoxia induced by noradrenaline. Lastly, because of the nature of the route of administration (i.e. intravenous vs. intrarenal arterial), it was not feasible for investigators to be blinded when they administered the treatments.

## 5 CONCLUSION

In conclusion, using a clinically relevant model of sepsis, we showed that renal arterial and intravenous infusions of tempol, during a clinically relevant therapeutic window, did not significantly attenuate renal medullary hypoxia in septic AKI. These observations in late stages of sepsis differed from our previous observations of attenuation of medullary hypoxia and restored renal function when the same dose of tempol was given intra‐arterially into the kidney in early sepsis. Together, they highlighted the importance of targeting the appropriate time point of sepsis progression for the development of effective therapeutic intervention in sepsis.

We have previously demonstrated that renal arterial infusion of tempol, administered at the onset of sepsis, prevented the development of renal medullary hypoxia and the development of AKI. However, we now show that the same intervention failed to confer reno‐protection when administered 24 h after sepsis onset, which is a time point that reflects when most patients begin treatment in intensive care settings. These findings underscore the need to prioritize drug development for later stages of sepsis and focus on intravenously administered therapies that are clinically feasible and that target multiple organs. In this context, our preclinical and clinical studies show that intravenous mega‐dose sodium ascorbate might represent a promising therapeutic strategy, because it reversed key pathophysiological features of sepsis, including renal medullary hypoxia and AKI, even when initiated at the clinically relevant 24 h mark of sepsis (Lankadeva et al., 2021; May et al., 2024; Yanase et al., 2023).

## AUTHOR CONTRIBUTIONS

Pei Chen Connie Ow, Rachel Peiris, Anton Trask‐Marino, Alemayehu Jufar, Ashenafi H Betrie, Adam Southon, Sally G. Hood and Abraham H Hulst were involved in acquisition, analysis, or interpretation of data. Clive N. May, R. Bellomo and Yugeesh R. Lankadeva were involved in conception and design of the work, acquisition and interpretation of data. All authors were involved in drafting of the work and revising it critically for important intellectual content. All authors approved the final version of the manuscript and agree to be accountable for all aspects of the work in ensuring that questions related to the accuracy or integrity of any part of the work are appropriately investigated and resolved. All persons designated as authors qualify for authorship, and all those who qualify for authorship are listed.

## CONFLICT OF INTEREST

None declared.

## Supporting information




**Figure S1**. Renal blood flow and vascular conductance during established sepsis, in response to 7 h treatment with either tempol or its vehicle and in the recovery period following resolution of sepsis with antibiotic.

**Figure S2**. Systemic haemodynamics during established sepsis, in response to 7 h treatment with either tempol or its vehicle and in the recovery period following resolution of sepsis with antibiotic.

**Table S1**. Determinants of renal tissue oxygenation in response to established sepsis and during 7 h treatment with either tempol or its vehicle.

**Table S2**. Determinants of renal tissue oxygenation in the recovery period following resolution of sepsis with antibiotic.

**Table S3**. Arterial blood gas and biochemistry during established sepsis and in response to 7 h treatment with either tempol or its vehicle.

**Table S4**. Arterial blood gas and biochemistry in the recovery period following resolution of sepsis with antibiotic.

## Data Availability

Data that support the findings of this study are available from the corresponding authors upon reasonable request.

## References

[eph70032-bib-0001] Basile, D. P. , Bonventre, J. V. , Mehta, R. , Nangaku, M. , Unwin, R. , Rosner, M. H. , Kellum, J. A. , & Ronco, C. (2016). Progression after AKI: Understanding maladaptive repair processes to predict and identify therapeutic treatments. Journal of the American Society of Nephrology, 27(3), 687–697. 26519085 10.1681/ASN.2015030309 PMC4769207

[eph70032-bib-0002] Bellomo, R. , Kellum, J. A. , Ronco, C. , Wald, R. , Martensson, J. , Maiden, M. , Bagshaw, S. M. , Glassford, N. J. , Lankadeva, Y. , Vaara, S. T. , & Schneider, A. (2017). Acute kidney injury in sepsis. Intensive Care Medicine, 43(6), 816–828. 28364303 10.1007/s00134-017-4755-7

[eph70032-bib-0003] Betrie, A. H. , Ma, S. , Ow, C. P. C. , Peiris, R. M. , Evans, R. G. , Ayton, S. , Lane, D. J. R. , Southon, A. , Bailey, S. R. , Bellomo, R. , May, C. N. , & Lankadeva, Y. R. (2023). Renal arterial infusion of tempol prevents medullary hypoperfusion, hypoxia, and acute kidney injury in ovine Gram‐negative sepsis. Acta Physiology, 239(1), e14025. 10.1111/apha.14025 PMC10909540 37548350

[eph70032-bib-0004] Calzavacca, P. , Evans, R. G. , Bailey, M. , Bellomo, R. , & May, C. N. (2015). Cortical and medullary tissue perfusion and oxygenation in experimental septic acute kidney injury. Critical Care Medicine, 43(10), e431–e439. 26181218 10.1097/CCM.0000000000001198

[eph70032-bib-0005] Calzavacca, P. , Evans, R. G. , Bailey, M. , Lankadeva, Y. R. , Bellomo, R. , & May, C. N. (2015). Long‐term measurement of renal cortical and medullary tissue oxygenation and perfusion in unanesthetized sheep. American Journal of Physiology‐Regulatory, Integrative and Comparative Physiology, 308(10), R832–R839. 25761701 10.1152/ajpregu.00515.2014

[eph70032-bib-0006] Chatterjee, P. K. , Cuzzocrea, S. , Brown, P. A. J. , Zacharowski, K. , Stewart, K. N. , Mota‐Filipe, H. , & Thiemermann, C. (2000). Tempol, a membrane‐permeable radical scavenger, reduces oxidant stress‐mediated renal dysfunction and injury in the rat. Kidney International, 58(2), 658–673. 10916089 10.1046/j.1523-1755.2000.00212.x

[eph70032-bib-0007] Eppel, G. A. , Bergström, G. , Anderson, W. P. , & Evans, R. G. (2003). Autoregulation of renal medullary blood flow in rabbits. American Journal of Physiology‐Regulatory, Integrative and Comparative Physiology, 284(1), R233–R244. 12388459 10.1152/ajpregu.00061.2002

[eph70032-bib-0008] Fleenor, B. S. , Seals, D. R. , Zigler, M. L. , & Sindler, A. L. (2012). Superoxide–lowering therapy with TEMPOL reverses arterial dysfunction with aging in mice. Aging Cell, 11(2), 269–276. 22168264 10.1111/j.1474-9726.2011.00783.x PMC3409251

[eph70032-bib-0009] Iguchi, N. , Kosaka, J. , Booth, L. C. , Iguchi, Y. , Evans, R. G. , Bellomo, R. , May, C. N. , & Lankadeva, Y. R. (2019). Renal perfusion, oxygenation, and sympathetic nerve activity during volatile or intravenous general anaesthesia in sheep. British Journal of Anaesthesia, 122(3), 342–349. 30770052 10.1016/j.bja.2018.11.018

[eph70032-bib-0010] Iguchi, N. , Kosaka, J. , Iguchi, Y. , Evans, R. G. , Bellomo, R. , May, C. N. , & Lankadeva, Y. R. (2020). Systemic haemodynamic, renal perfusion and renal oxygenation responses to changes in inspired oxygen fraction during total intravenous or volatile anaesthesia. British Journal of Anaesthesia, 125(2), 192–200. 32563492 10.1016/j.bja.2020.03.033

[eph70032-bib-0011] Ishikawa, K. , Calzavacca, P. , Bellomo, R. , Bailey, M. , & May, C. N. (2012). Effect of selective inhibition of renal inducible nitric oxide synthase on renal blood flow and function in experimental hyperdynamic sepsis. Critical Care Medicine, 40(8), 2368–2375. 22622397 10.1097/CCM.0b013e3182514be9

[eph70032-bib-0012] Langenberg, C. , Gobe, G. , Hood, S. , May, C. N. , & Bellomo, R. (2014). Renal histopathology during experimental septic acute kidney injury and recovery. Critical Care Medicine, 42(1), e58–e67. 24126439 10.1097/CCM.0b013e3182a639da

[eph70032-bib-0013] Langenberg, C. , Wan, L. , Egi, M. , May, C. N. , & Bellomo, R. (2007). Renal blood flow and function during recovery from experimental septic acute kidney injury. Intensive Care Medicine, 33(9), 1614–1618. 17572879 10.1007/s00134-007-0734-8

[eph70032-bib-0014] Lankadeva, Y. R. , Kosaka, J. , Evans, R. G. , Bailey, S. R. , Bellomo, R. , & May, C. N. (2016). Intrarenal and urinary oxygenation during norepinephrine resuscitation in ovine septic acute kidney injury. Kidney International, 90(1), 100–108. 27165831 10.1016/j.kint.2016.02.017

[eph70032-bib-0015] Lankadeva, Y. R. , Kosaka, J. , Evans, R. G. , Bellomo, R. , & May, C. N. (2018). Urinary oxygenation as a surrogate marker of medullary oxygenation during angiotensin II therapy in septic acute kidney injury. Critical Care Medicine, 46(1), e41–e48. 29077618 10.1097/CCM.0000000000002797

[eph70032-bib-0016] Lankadeva, Y. R. , Kosaka, J. , Evans, R. G. , & May, C. N. (2018). An ovine model for studying the pathophysiology of septic acute kidney injury. In B. Tharakan (Ed.). Traumatic and ischemic injuries: Methods and protocols (pp. 207–18), 1717. 10.1007/978-1-4939-7526-6_16 29468594

[eph70032-bib-0017] Lankadeva, Y. R. , Peiris, R. M. , Okazaki, N. , Birchall, I. E. , Trask‐Marino, A. , Dornom, A. , Vale, T. A. M. , Evans, R. G. , Yanase, F. , Bellomo, R. , & May, C. N. (2021). Reversal of the pathophysiological responses to Gram‐negative sepsis by megadose vitamin C. Critical Care Medicine, 49(2), e179–e190. 33239507 10.1097/CCM.0000000000004770 PMC7803449

[eph70032-bib-0018] Lerolle, N. , Nochy, D. , Guérot, E. , Bruneval, P. , Fagon, J.‐Y. , Diehl, J.‐L. , & Hill, G. (2010). Histopathology of septic shock induced acute kidney injury: Apoptosis and leukocytic infiltration. Intensive Care Medicine, 36(3), 471–478. 19924395 10.1007/s00134-009-1723-x

[eph70032-bib-0019] Liu, B. , Hu, Y. , Tian, D. , Dong, J. , & Li, B.‐F. (2021). Assessing the effects of tempol on renal fibrosis, inflammation, and oxidative stress in a high‐salt diet combined with 5/6 nephrectomy rat model: Utilizing oxidized albumin as a biomarker. BioMed Central Nephrology [Electronic Resource], 25(1), 64. 10.1186/s12882-024-03495-0 PMC10893674 38395806

[eph70032-bib-0020] Liu, J. , Xie, H. , Ye, Z. , Li, F. , & Wang, L. (2020). Rates, predictors, and mortality of sepsis‐associated acute kidney injury: A systematic review and meta‐analysis. BioMed Central Nephrology [Electronic Resource], 21(1), 318. 32736541 10.1186/s12882-020-01974-8 PMC7393862

[eph70032-bib-0021] Luan, J. , Li, W. , Han, J. , Zhang, W. , Gong, H. , & Ma, R. (2012). Renal protection of in vivo administration of tempol in streptozotocin‐induced diabetic rats. Journal of Pharmacological Sciences, 119(2), 167–176. 22673147 10.1254/jphs.12002fp PMC3539787

[eph70032-bib-0022] Ludbrook, J. (1994). Repeated measures and multiple comparisons in cardiovascular research. Cardiovascular Research, 28(3), 303–311. 8174149 10.1093/cvr/28.3.303

[eph70032-bib-0023] Maiden, M. J. , Otto, S. , Brealey, J. K. , Finnis, M. E. , Chapman, M. J. , Kuchel, T. R. , Nash, C. H. , Edwards, J. , & Bellomo, R. (2016). Structure and function of the kidney in septic shock. A prospective controlled experimental study. American Journal of Respiratory and Critical Care Medicine, 194(6), 692–700. 26967568 10.1164/rccm.201511-2285OC

[eph70032-bib-0024] May, C. N. , Ow, C. P. , Pustovit, R. V. , Lane, D. J. , Jufar, A. H. , Trask‐Marino, A. , Peiris, R. M. , Gunn, A. , Booth, L. C. , Plummer, M. P. , Bellomo, R. , & Lankadeva, Y. R. (2024). Reversal of cerebral ischaemia and hypoxia and of sickness behaviour by megadose sodium ascorbate in ovine Gram‐negative sepsis. British Journal of Anaesthesia, 133(2), 316–325. 38960833 10.1016/j.bja.2024.04.058

[eph70032-bib-0025] Osawa, E. A. , Cutuli, S. L. , Bitker, L. , Canet, E. , Cioccari, L. , Iguchi, N. , Lankadeva, Y. R. , Eastwood, G. M. , Evans, R. G. , May, C. N. , & Bellomo, R. (2019). Effect of furosemide on urinary oxygenation in patients with septic shock. Blood Purification, 48(4), 336–345. 31336370 10.1159/000501512

[eph70032-bib-0026] Ow, C. P. C. , Ngo, J. P. , Ullah, M. M. , Hilliard, L. M. , & Evans, R. G. (2018). Renal hypoxia in kidney disease: Cause or consequence? Acta Physiology, 222(4), e12999. 10.1111/apha.12999 29159875

[eph70032-bib-0027] Percie Du Sert, N. , Hurst, V. , Ahluwalia, A. , Alam, S. , Avey, M. T. , Baker, M. , Browne, W. J. , Clark, A. , Cuthill, I. C. , Dirnagl, U. , Emerson, M. , Garner, P. , Holgate, S. T. , Howells, D. W. , Karp, N. A. , Lazic, S. E. , Lidster, K. , Maccallum, C. J. , Macleod, M. , …, Würbel, H. (2020). The ARRIVE guidelines 2.0: Updated guidelines for reporting animal research. PLoS Biology, 18(7), e3000410. 32663219 10.1371/journal.pbio.3000410 PMC7360023

[eph70032-bib-0028] Plummer, M. P. , Lankadeva, Y. R. , Finnis, M. E. , Harrois, A. , Harding, C. , Peiris, R. M. , Okazaki, N. , May, C. N. , Evans, R. G. , Macisaac, C. M. , Barge, D. , Bellomo, R. , & Deane, A. M. (2021). Urinary and renal oxygenation during dexmedetomidine infusion in critically ill adults with mechanistic insights from an ovine model. Journal of Critical Care, 64, 74–81. 33794470 10.1016/j.jcrc.2021.03.004

[eph70032-bib-0029] Rudd, K. E. , Johnson, S. C. , Agesa, K. M. , Shackelford, K. A. , Tsoi, D. , Kievlan, D. R. , Colombara, D. V. , Ikuta, K. S. , Kissoon, N. , Finfer, S. , Fleischmann‐Struzek, C. , Machado, F. R. , Reinhart, K. K. , Rowan, K. , Seymour, C. W. , Watson, R. S. , West, T. E. , Marinho, F. , Hay, S. I. , …, Naghavi, M. (2020). Global, regional, and national sepsis incidence and mortality, 1990–2017: Analysis for the Global Burden of Disease Study. The Lancet, 395(10219), 200–211. 10.1016/S0140-6736(19)32989-7 PMC6970225 31954465

[eph70032-bib-0030] Welch, W. J. , & Wilcox, S. C. (2001). AT1 receptor antagonist combats oxidative stress and restores nitric oxide signaling in the SHR. Kidney International, 59(4), 1257–1263. 11260386 10.1046/j.1523-1755.2001.0590041257.x

[eph70032-bib-0031] Wilcox, S. C. (2010). Effects of tempol and redox‐cycling nitroxides in models of oxidative stress. Pharmacology & Therapeutics, 126(2), 119–145. 20153367 10.1016/j.pharmthera.2010.01.003 PMC2854323

[eph70032-bib-0032] Wilcox, S. C. , & Pearlman, A. (2008). Chemistry and antihypertensive effects of tempol and other nitroxides. Pharmacological Reviews, 60(4), 418–469. 19112152 10.1124/pr.108.000240 PMC2739999

[eph70032-bib-0033] Work Group Membership . (2012). KDIGO clinical practice guideline for acute kidney injury. Kidney International Supplements, 2, 1–138.

[eph70032-bib-0034] Yanase, F. , Spano, S. , Maeda, A. , Chaba, A. , Naorungroj, T. , Ow, C. P. C. , Lankadeva, Y. R. , May, C. N. , Betrie, A. H. , Lane, D. J. R. , Eastwood, G. M. , Plummer, M. P. , & Bellomo, R. (2023). Mega‐dose sodium ascorbate: A pilot, single‐dose, physiological effect, double‐blind, randomized, controlled trial. Critical Care (London, England), 27(1), 371. 37828547 10.1186/s13054-023-04644-x PMC10571252

[eph70032-bib-0035] Zarbock, A. , Nadim, M. K. , Pickkers, P. , Gomez, H. , Bell, S. , Joannidis, M. , Kashani, K. , Koyner, J. L. , Pannu, N. , Meersch, M. , Reis, T. , Rimmelé, T. , Bagshaw, S. M. , Bellomo, R. , Cantaluppi, V. , Deep, A. , De Rosa, S. , Perez‐Fernandez, X. , Husain‐Syed, F. , … Forni, L. G. (2023). Sepsis‐associated acute kidney injury: Consensus report of the 28th acute disease quality initiative workgroup. Nature Reviews Nephrology, 19(6), 401–417. 36823168 10.1038/s41581-023-00683-3

