# Supplementary figures and images for: Effects of tempol on renal medullary tissue hypoxia in an ovine model of Gram‐negative septic acute kidney injury
Source: Exp Physiol. 2025 Sep 22:10.1113/EP092396. Online ahead of print. doi: 10.1113/EP092396 (PMC13394757; doi:10.1113/EP092396)

Suppl Fig 1

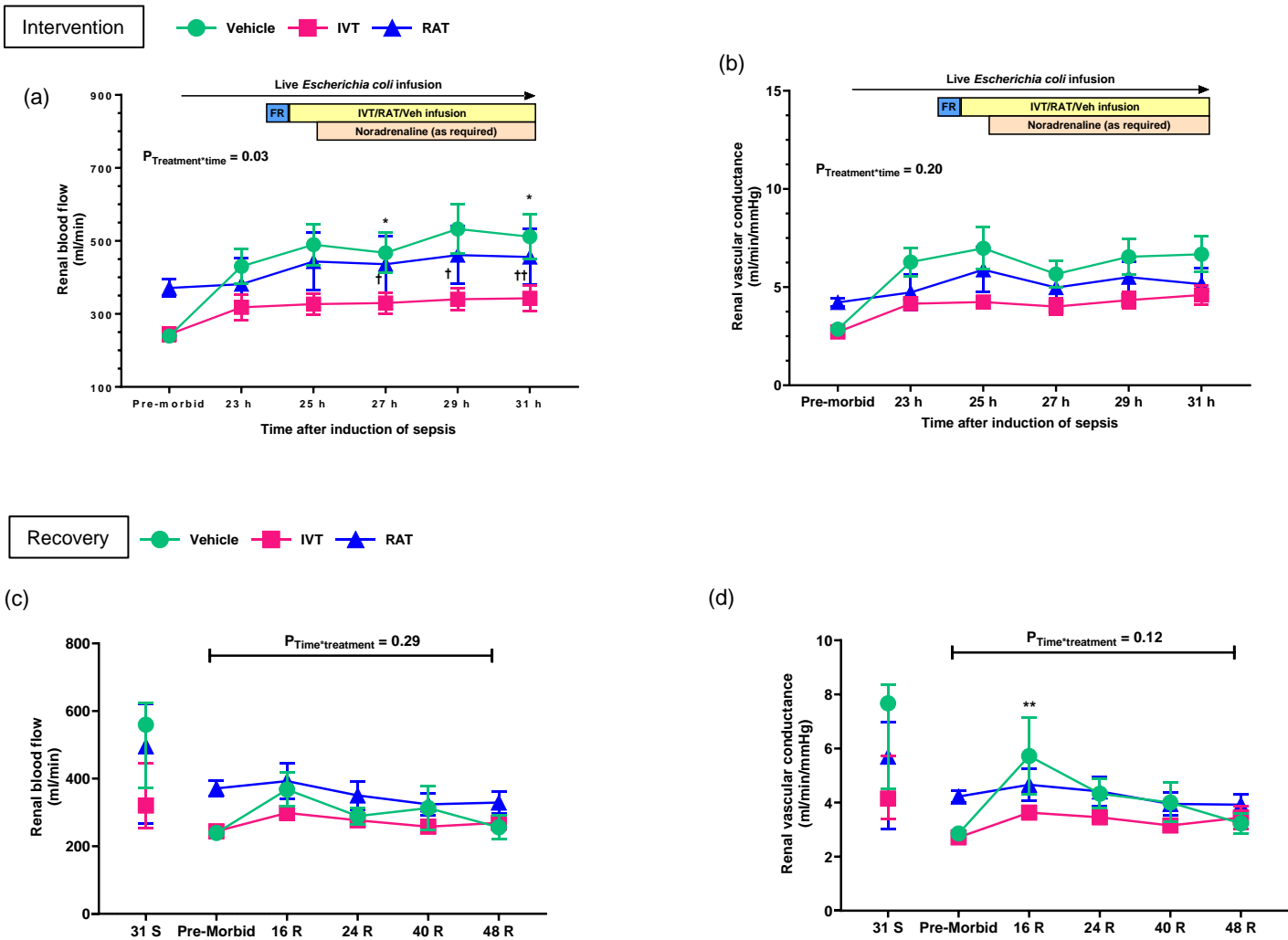

Supplement: Supplementary file 1 — Figure S1. Renal blood flow and vascular conductance during established sepsis, in response to 7 h treatment with either tempol or its vehicle and in the recovery period following resolution of sepsis with antibiotic. [file EPH-9999-0-s003.pdf]

Suppl Fig 2

Intervention      ● Vehicle      ■ IVT      ▲ RAT

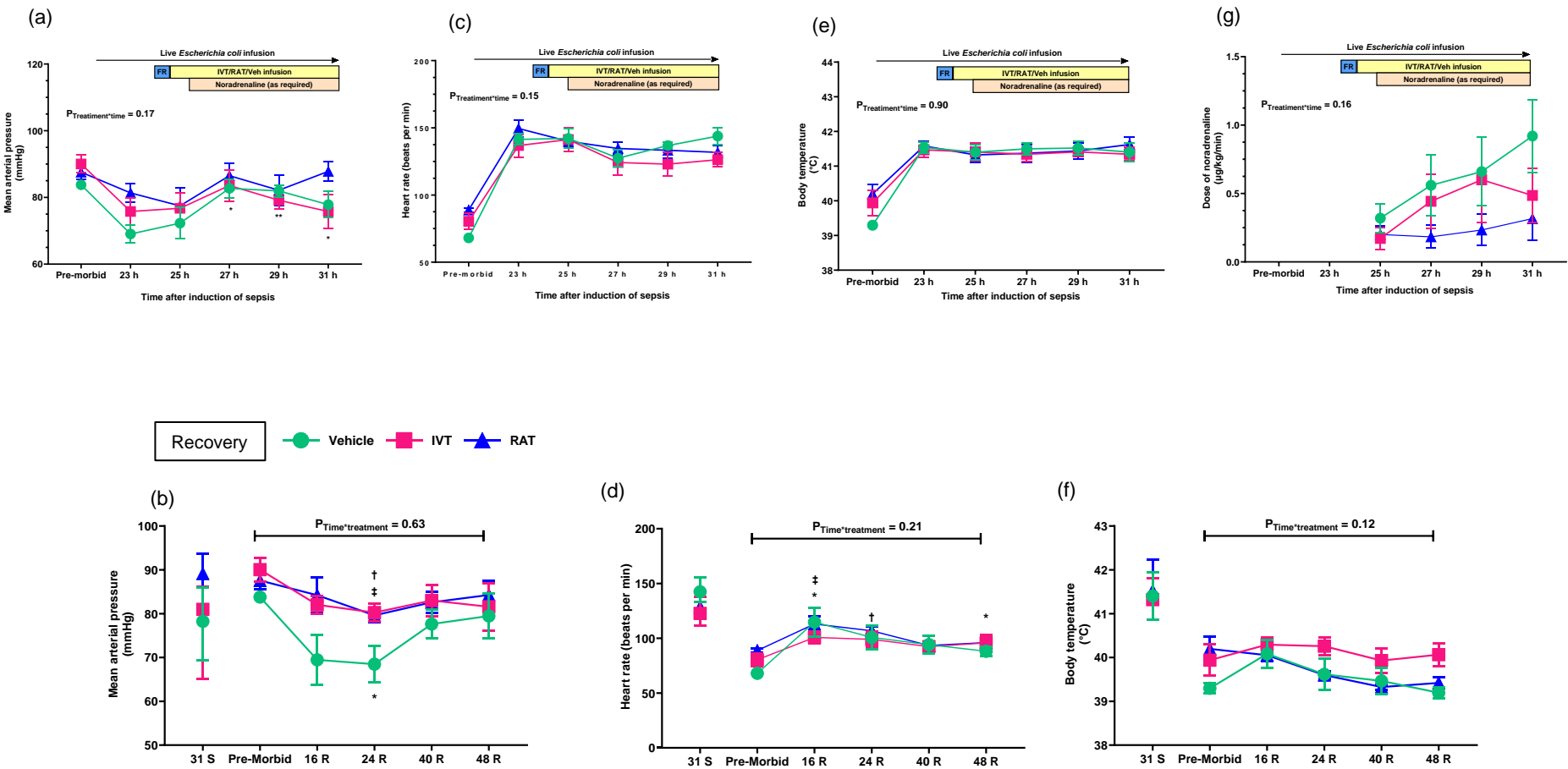

Supplement: Supplementary file 2 — Figure S2. Systemic haemodynamics during established sepsis, in response to 7 h treatment with either tempol or its vehicle and in the recovery period following resolution of sepsis with antibiotic. [file EPH-9999-0-s002.pdf]
