# Supplementary material for: Effects of tempol on renal medullary tissue hypoxia in an ovine model of Gram‐negative septic acute kidney injury
Source: Exp Physiol. 2025 Sep 22:10.1113/EP092396. Online ahead of print. doi: 10.1113/EP092396 (PMC13394757; doi:10.1113/EP092396)
Supplement: Supplementary file 3 — Table S1. Determinants of renal tissue oxygenation in response to established sepsis and during 7 h treatment with either tempol or its vehicle. [file EPH-9999-0-s004.pdf]

Suppl Table 1

Variable	Treatment	Experimental time point						Two-way RM ANOVA
				Intervention				
		Pre-morbid baseline	23 h sepsis	25 h sepsis	27 h sepsis	29 h sepsis	31 h sepsis	$P_{treatment*time}$
Renal DO_2 (mL O_2/min)	Vehicle	31.9 ± 3.1	57.8 ± 7.9	55.4 ± 6.1	56.0 ± 8.0	63.3 ± 8.5	61.7 ± 7.8	0.26
	IVT	32.8 ± 3.4	44.2 ± 6.2	41.0 ± 5.2	41.5 ± 5.5	43.2 ± 5.3	42.8 ± 5.9	
	RAT	43.8 ± 3.0	48.5 ± 8.3	49.6 ± 8.7	50.1 ± 8.4	53.0 ± 8.8	53.0 ± 8.3	
Renal VO_2 (mL O_2/min)	Vehicle	3.2 ± 0.4	4.3 ± 0.8	4.1 ± 0.9	2.5 ± 0.6	3.4 ± 0.9	3.5 ± 0.7	0.27
	IVT	3.0 ± 0.5	3.3 ± 0.4	4.3 ± 0.4	3.7 ± 0.4	3.0 ± 0.1	3.2 ± 0.4	
	RAT	5.2 ± 0.3	4.6 ± 0.8	5.2 ± 0.7	5.1 ± 0.8	5.0 ± 0.9	4.5 ± 1.1	
Renal extraction of O_2 (%)	Vehicle	10.7 ± 0.9	7.7 ± 1.9	7.9 ± 2.6	4.6 ± 1.2	5.8 ± 2.3	5.9 ± 1.8	0.5
	IVT	10.6 ± 0.5	9.2 ± 2.4	12.0 ± 2.1	9.5 ± 2.3	8.0 ± 2.3	8.7 ± 1.5	
	RAT	11.4 ± 1.4	7.7 ± 1.8	8.8 ± 1.8	8.4 ± 1.8	7.9 ± 1.6	7.1 ± 2.0	
