# Supplementary material for: Effects of tempol on renal medullary tissue hypoxia in an ovine model of Gram‐negative septic acute kidney injury
Source: Exp Physiol. 2025 Sep 22:10.1113/EP092396. Online ahead of print. doi: 10.1113/EP092396 (PMC13394757; doi:10.1113/EP092396)
Supplement: Supplementary file 4 — Table S2. Determinants of renal tissue oxygenation in the recovery period following resolution of sepsis with antibiotic. [file EPH-9999-0-s005.pdf]

Suppl Table 2

Variable	Treatment	Experimental time point						Two-way RM ANOVA
				Resolution of sepsis with antibiotic treatment				
		Pre-morbid baseline	31 h sepsis	16 h recovery	24 h recovery	40 h recovery	48 h recovery	$P_{treatment*time}$
Renal DO_2 (ml O_2/min)	Vehicle	30.2 (27.7, 34.6)	57.8 ± 7.9	55.6 (43.3, 77.6)	60.2 (46.9, 87.7)	80.6 (46.8, 92.3)	81.3 (46.7, 90.4)	0.04
	IVT	35.7 (25.1, 40.2)	44.2 ± 6.2	35.9 (32.7, 44.7)	45.2 (32.4, 54.0)	45.5 (29.7, 56.8)	52.2 (29.1, 57.4)	
	RAT	43.4 (37.1, 51.0)	48.5 ± 8.3	62.4 (26.5, 73.1)	63.2 (30.5, 71.1)	64.7 (34.3, 75.1)	63.6 (36.3, 75.0)	
Renal VO_2 (ml O_2/min)	Vehicle	3.3 ± 0.4	4.3 ± 0.8	4.9 ± 0.7	5.7 ± 0.5	8.8 ± 1.0	8.0 ± 2.1	0.22
	IVT	3.0 ± 0.5	3.3 ± 0.4	4.4 ± 0.9	4.4 ± 1.2	5.6 ± 1.2	6.3 ± 1.0	
	RAT	5.3 ± 0.2	4.6 ± 0.8	5.6 ± 1.1	6.7 ± 1.3	6.4 ± 1.5	7.0 ± 1.6	
Renal extraction of O_2 (%)	Vehicle	10.7 ± 1.0	7.7 ± 1.9	7.8 ± 1.1	8.2 ± 0.5	11.7 ± 1.0	9.8 ± 2.1	0.21
	IVT	10.6 ± 0.5	9.2 ± 2.4	10.1 ± 0.6	9.0 ± 1.5	13.3 ± 1.8	13.0 ± 1.0	
	RAT	11.7 ± 1.2	7.7 ± 1.8	9.3 ± 0.8	11.1 ± 1.0	10.0 ± 1.1	11.0 ± 1.2	
