# Supplementary material for: Effects of tempol on renal medullary tissue hypoxia in an ovine model of Gram‐negative septic acute kidney injury
Source: Exp Physiol. 2025 Sep 22:10.1113/EP092396. Online ahead of print. doi: 10.1113/EP092396 (PMC13394757; doi:10.1113/EP092396)
Supplement: Supplementary file 5 — Table S3. Arterial blood gas and biochemistry during established sepsis and in response to 7 h treatment with either tempol or its vehicle. [file EPH-9999-0-s001.pdf]

Suppl Table 3

Variable	Treatment	Experimental time point						Two-way RM ANOVA
				Intervention period				
		Premorbid baseline	23 h sepsis	25 h sepsis	27 h sepsis	29 h sepsis	31 h sepsis	$P_{treatment*time}$
pH	Vehicle	7.53 (7.50, 7.53)	7.57 (7.48, 7.57)	7.49 (7.46, 7.54)	7.54 (7.48, 7.56)	7.53 (7.51, 7.55)	7.53 (7.52, 7.56)	0.013
	IVT	7.50 (7.49, 7.53)	7.56 (7.55, 7.60)	7.54 (7.52, 7.55)	7.51 (7.50, 7.54)	7.52 (7.49, 7.53)	7.53 (7.48, 7.57)	
	RAT	7.54 (7.51, 7.56)	7.53 (7.47, 7.55)	7.49 (7.46, 7.54)	7.54 (7.48, 7.56)	7.53 (7.51, 7.55)	7.53 (7.52, 7.56)	
pO_2 (mmHg)	Vehicle	109.1 (103.7, 112. 8)	83.9 (69.3, 88.6)	78.5 (73.4, 87.5)	73.5 (62.0, 84.5)	72.8 (69.1, 91.3)	76.2 (74.7, 87.8)	0.73
	IVT	97.3 (89.4, 108.2)	91.3 (81.5, 96.2)	83.0 (79.8, 103.7)	86.9 (78.5, 94.6)	88.6 (81.6, 94.9)	88.9 (84.5, 99.1)	
	RAT	110.6 (101.3, 119.2)	94.2 (83.7, 121.0)	78.5 (73.4, 87.5)	73.5 (62.0, 84.5)	72.8 (69.1, 91.3)	76.2 (74.7, 87.8)	
pCO_2 (mmHg)	Vehicle	32.7 ± 1.0	30.5 ± 1.2	36.0 ± 3.2	34.5 ± 3.2	32.9 ± 2.8	33.3 ± 2.4	0.20
	IVT	33.5 ± 1.5	28.8 ± 1.9	31.3 ±1.0	31.0 ± 1.6	31.3 ± 1.6	30.4 ± 1.7	
	RAT	30.6 ± 1.1	30.3 ± 1.7	30.5 ± 3.2	30.6 ± 3.2	29.5 ± 1.4	31.6 ± 2.0	
SO_2 (%)	Vehicle	97.6 ± 0.4	93.6 ± 2.7	94.7 ± 1.1	92.8 ± 2.3	95.1 ± 1.1	96.0 ± 0.7	0.06
	IVT	96.3 ± 0.8	96.7 ± 0.3	95.8 ± 0.9	96.0 ± 0.6	95.6 ± 0.7	96.3 ± 0.6	
	RAT	97.4 ± 0.4	95.6 ± 1.4	95.3 ± 1.4	95.6 ± 1.3	95.6 ± 1.3	95.0 ± 1.7	
HCO_3^- (mmol/L)	Vehicle	26.2 ± 0.4	28.4 ± 1.2	27.2 ± 1.2	28.1 ± 1.5	26.8 ± 1.8	28.1 ± 1.8	0.06
	IVT	26.5 ± 0.9	25.8 ± 1.0	26.4 ± 1.0	25.1 ± 1.2	25.1 ± 1.1	25.3 ± 1.2	
	RAT	26.5 ± 0.8	23.9 ± 0.8 [#]	22.8 ± 1.1 [#]	24.3 ± 0.5 [#]	24.5 ± 0.6	25.8 ± 0.3	
Na^+ (mmol/L)	Vehicle	140.6 ± 0.8	138.8 ± 0.7	138.6 ± 1.5	139.2 ± 1.3	138.4 ± 1.1	137.6 ± 1.2	0.82
	IVT	139.0 ± 1.9	135.4 ± 1.0	136.7 ± 1.3 [$$]	136.6 ± 1.1 [$]	138.0 ± 0.8 [$$]	133.9 ± 3.2	
	RAT	133.7 ± 1.3	130.3 ± 2.1 [#]	130.0 ± 1.0 [##]	130.5 ± 1.5 [##]	131.2 ± 1.0 [##]	131.5 ± 1.8 [#]	
Lactate (mmol/L)	Vehicle	0.5 ± 0.1	2.0 ± 0.4	3.0 ± 0.4 [*]	2.2 ± 0.6	2.2 ± 0.6	1.8 ± 0.5	0.09
	IVT	0.5 ± 0.1	1.1 ± 0.2	1.4 ± 0.3	1.1 ± 0.2	1.4 ± 0.3	0.9 ± 0.2	
	RAT	0.7 ± 0.1	1.4 ± 0.3	2.6 ± 0.5	1.6 ± 0.4	1.1 ± 0.2	1.0 ± 0.2	
Hemoglobin (g/dL)	Vehicle	10.2 (8.9, 10.9)	10.1 (8.5, 12.1)	8.1 (7.5, 10.2) [*]	8.7 (8.2, 10.9)	8.5 (7.9, 10.7)	8.9 (7.8, 10.4)	0.44
	IVT	9.3 (8.8, 10.4)	9.4 (9.3, 11.1)	8.8 (7.6, 10.6) [‡‡]	9.0 (8.0, 10.4) [‡‡]	9.2 (8.6, 10.1) [‡]	8.8 (8.5, 10.0) [‡‡]	
	RAT	8.4 (9.0, 9.6)	9.7 (8.3, 11.5)	8.8 (6.7, 10.1) [†]	8.9 (7.1, 10.3) [†]	8.9 (7.1, 10.1) [††]	8.9 (7.6, 10.1) [††]	
