# Supplementary material for: Effects of tempol on renal medullary tissue hypoxia in an ovine model of Gram‐negative septic acute kidney injury
Source: Exp Physiol. 2025 Sep 22:10.1113/EP092396. Online ahead of print. doi: 10.1113/EP092396 (PMC13394757; doi:10.1113/EP092396)
Supplement: Supplementary file 6 — Table S4. Arterial blood gas and biochemistry in the recovery period following resolution of sepsis with antibiotic. [file EPH-9999-0-s006.pdf]

Suppl Table 4

Variable	Treatment	Experimental time point						Two-way RM ANOVA
				Resolution of sepsis with antibiotic treatment				
		Pre-morbid	31 h sepsis	16 h recovery	24 h recovery	40 h recovery	48 h recovery	$P_{treatment*time}$
pH	Vehicle	7.52 ± 0.01	7.57 (7.48, 7.57)	7.52 ± 0.02	7.51 ± 0.01	7.50 ± 0.01	7.49 ± 0.02	0.21
	IVT	7.51 ± 0.01	7.56 (7.55, 7.60)	7.53 ± 0.01	7.51 ± 0.01	7.51 ± 0.01	7.49 ± 0.01	
	RAT	7.53 ± 0.02	7.53 (7.47, 7.55)	7.50 ± 0.01	7.48 ± 0.01	7.49 ± 0.01	7.46 ± 0.02	
pO_2 (mmHg)	Vehicle	108.4 ± 2.1	83.9 (69.3, 88.6)	88.5 ± 6.7	93.0 ± 4.6	95.6 ± 6.1	103.4 ± 5.1	0.41
	IVT	97.9 ± 4.0	91.3 (81.5, 96.2)	89.3 ± 3.2	98.6 ± 5.0	97.1 ± 4.9	93.1 ± 3.9	
	RAT	111.7 ± 4.6	94.2 (83.7, 121.0)	100.2 ± 6.5	112.8 ± 10.8	102.2 ± 4.1	105.3 ± 5.2	
pCO_2 (mmHg)	Vehicle	32.7 ± 1.0	30.5 ± 1.2	35.9 ± 1.4	36.1 ± 1.0	34.1 ± 1.1	33.5 ± 1.7	0.13
	IVT	33.5 ± 1.5	28.8 ± 1.9	32.0 ± 0.5	32.3 ± 1.7	30.3 ± 1.3	33.4 ± 1.2	
	RAT	30.6 ± 1.1	30.3 ± 1.7	34.2 ± 2.1	35.4 ± 1.1	32.9 ± 0.9	34.2 ± 1.2	
SO_2 (%)	Vehicle	97.6 ± 0.4	93.6 ± 2.7	95.6 ± 1.0	96.5 ± 0.8	96.8 ± 0.5	96.2 ± 0.9	0.54
	IVT	96.3 ± 0.8	96.7 ± 0.3	95.9 ± 0.8	96.8 ± 0.5	96.9 ± 0.3	96.2 ± 0.3	
	RAT	97.4 ± 0.4	95.6 ± 1.4	95.9 ± 1.0	96.6 ± 0.8	96.6 ± 0.5	96.3 ± 1.9	
HCO_3^- (mmol/L)	Vehicle	26.2 ± 0.4	28.4 ± 1.2	29.7 ± 1.6	28.9 ± 1.3	26.6 ± 1.0	25.3 ± 1.0	0.004
	IVT	26.5 ± 0.9	25.8 ± 1.0	26.2 ± 0.6	25.5 ± 1.4	25.1 ± 1.2	25.4 ± 1.4	
	RAT	26.5 ± 0.8	23.9 ± 0.8 #	26.4 ± 1.4	26.1 ± 1.2	25.0 ± 0.8	23.1 ± 1.0	
Na^+ (mmol/L)	Vehicle	140.6 ± 0.8	138.8 ± 0.7	138.6 ± 0.6	136.8 ± 2.2	136.2 ± 0.8	135.6 ± 1.1	0.16
	IVT	139.0 ± 1.9	135.4 ± 1.0	136.6 ± 1.2	135.6 ± 1.4	137.9 ± 0.6	139.1 ± 1.1	
	RAT	133.7 ± 1.3	130.3 ± 2.1 #	134.5 ± 3.0	132.8 ± 2.3	133.3 ± 1.4	134.8 ± 1.4	
Lactate (mmol/L)	Vehicle	0.52 ± 0.09	2.0 ± 0.4	0.70 ± 0.10	0.48 ± 0.05	0.44 ± 0.04	0.36 ± 0.02	0.01
	IVT	0.49 ± 0.06	1.1 ± 0.2	0.46 ± 0.02	0.37 ± 0.03	0.47 ± 0.08	0.40 ± 0.04	
	RAT	0.73 ± 0.13	1.4 ± 0.3	0.48 ± 0.05	0.38 ± 0.03	0.45 ± 0.09	0.45 ± 0.09	
Hemoglobin (g/dL)	Vehicle	10.1 ± 0.8	10.1 (8.5, 12.1)	10.1 ± 0.7	9.9 ± 0.6	10.1 ± 0.5	9.7 ± 0.5	0.30
	IVT	9.5 ± 0.4	9.4 (9.3, 11.1)	9.0 ± 0.4	9.5 ± 0.5	9.4 ± 0.6	9.6 ± 0.6	
	RAT	8.6 ± 0.3	9.7 (8.3, 11.5)	9.0 ± 0.5	8.9 ± 0.4	9.2 ± 0.3	9.4 ± 0.3	
